# The impact of surgical experience and frequency of practice on perioperative outcomes in pancreatic surgery

**DOI:** 10.1186/s12893-019-0577-6

**Published:** 2019-08-13

**Authors:** Christian Krautz, Elisabeth Haase, Moustafa Elshafei, Hans-Detlev Saeger, Marius Distler, Robert Grützmann, Georg F. Weber

**Affiliations:** 1 Klinik für Allgemein- und Viszeralchirurgie, Friedrich-Alexander-Universität Erlangen-Nürnberg, Universitätsklinikum Erlangen, Krankenhausstrasse 12, 91054 Erlangen, Germany; 2 Klinik für Viszeral-, Thorax- und Gefäßchirurgie, Technische Universität Dresden, Universtitätsklinikum Dresden, Fetscherstrasse 74, 01307 Dresden, Germany

**Keywords:** In-hospital mortality, Frequency of practice, Pancreatic surgery, Perioperative outcomes, Surgical experience, Volume–outcome relationship

## Abstract

**Objective:**

We aimed to determine the impact of surgical experience and frequency of practice on perioperative morbidity and mortality in pancreatic surgery.

**Methods:**

1281 patients that underwent pancreatic resections from 1993 to 2013 were retrospectively analyzed using logistic regression models. All cases were stratified according to the surgeon’s level of experience, which was based on the number of previously performed pancreatic resections and the extent of received supervision (novice: *n* <  20 / intensive; intermediate: *n* = 21–90 / decreasing; and experienced surgeon: *n* > 90 / none). Additional stratification was based on the frequency of practice (sporadic: 3 resections > 6 weeks, frequent: 3 resections ≤6 weeks).

**Results:**

The novice and experienced categories were related to a decreased risk of postoperative pancreatic fistulas (odds ratio [OR] 0.46, 95% confidence interval [CI] 0.26–0.82 and 0.54, 95% CI 0.36–0.82) and in-hospital mortality (OR 0.45, 95% CI 0.17–1.16 and 0.42, 95% CI 0.21–0.83) compared to the intermediate category. Frequent practice was associated with a significantly lower risk of delayed gastric emptying (OR 0.56, 95% CI 0.38–0.83), postpancreatectomy hemorrhage (OR 0.64, 95% CI 0.42–0.98) and in-hospital mortality (OR 0.45, 95% CI 0.24–0.87).

**Conclusions:**

Our results emphasize the importance of supervision within a pancreatic surgery training program. In addition, our data underline the need of a sufficient patient caseload to ensure frequent practice.

## Background

Several studies have found an inverse relationship between hospital volume and perioperative outcomes in complex surgery [1–4]. There is evidence that such associations also exist between the annual surgeon volume and operative mortality [5–7]. A population-based study examining the level of surgical skill and clinical outcomes in bariatric surgery found that greater skill was associated with fewer postoperative complications [8]. The growing body of outcomes research has yielded to changes in health care that are indirectly interfering with the traditional model of surgical training in general surgery. Consequently, a debate regarding the best manner for surgeons to acquire surgical competency in an increasingly outcome-oriented environment has been triggered. Concurrently, in recent years, there has been a paradigm shift in surgical education, with a movement of the acquisition phase of surgical skills from the operation room to the surgical skills laboratory [9]. But achieving competence in complex pancreatic surgery is a demanding process that involves surgical skills that cannot be acquired by surgical simulation. Therefore, surgical training in pancreatic surgery is still dependent on the traditional model of surgical training through apprenticeship [10]. Several studies have examined the impact of surgical experience on perioperative outcomes or explored the existence of a learning curve in pancreatic surgery [7, 11–13]. However, the impact of surgical experience and frequency of practice on perioperative outcomes has not been sufficiently evaluated in the context of pancreatic surgery training.

To address this issue, we conducted a retrospective study of all cases of pancreatic resections stratified according to the operating surgeon’s level of experience and frequency of practice in pancreatic surgery. We postulated three hypotheses for the current study of pancreatic surgery training: 1) Stringent case selection and intensive supervision will result in comparable postoperative morbidity of novices and experienced surgeons. 2) Reduction of supervision and less stringent case selection will impair outcomes. 3) Frequent practice of pancreatic resections will improve perioperative outcomes.

## Methods

### Data

We retrospectively analyzed patients who underwent pancreatic resections from 1993 to 2013 at the Department of Visceral, Thoracic and Vascular Surgery of the University Hospital Dresden. We used the institutional database that included information on the composition of the surgical team (e.g. operating surgeon and assisting surgeon). From 1993 to 2013, only surgeons that passed residency training for general surgery were eligible to start pancreatic surgery training. The pancreatic surgery training curriculum mandated full-time supervision by an experienced surgeon and patient cases of low surgical difficulty for at least 20 resections. Thereafter, supervision was decreased according to the trainees’ proficiency and the surgical difficulty of the individual patient case.

In accordance with the guidelines for human subject research, approval was obtained from the ethics committee at the Carl Gustav Carus University Hospital (decision number EK 404102018).

### Definition of surgical experience and frequency of practice

**Fig. 1:**
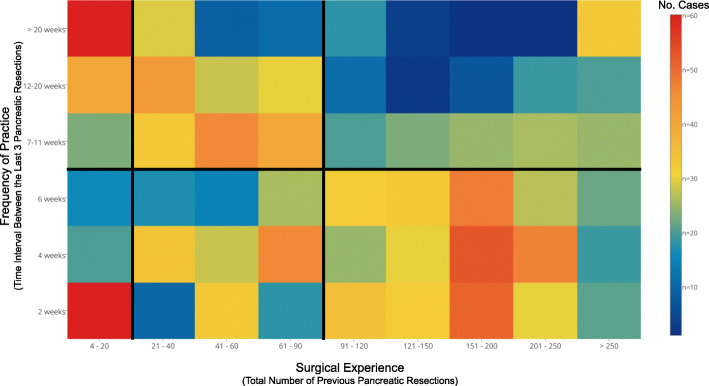
Heat map of Case Distribution According to the Surgeon’s Level of Experience and Frequency of Practice

Prior to the statistical analysis, we generated a heat map that cross-examined the distribution of all cases according to the time interval between the last three resections and the total number of previously performed pancreatic resections of the operating surgeon (Fig. 1 in Appendix). This heat map was used to determine the definitions of surgical experience and frequency of practice, as we assumed that an increasing experience will essentially yield in a higher rate of surgical case assignment and vice versa.

Subsequently, each operative case was stratified according to the operating surgeon’s experience level and frequency of practice in pancreatic surgery at the time of resection (Table 6 in Appendix). Pancreatic surgery novices were defined as surgeons with a total of less than 20 pancreatic resections, as predetermined by the training curriculum of our department. Intermediate experienced surgeons were those who had performed their 21st - 90th pancreatic resection. With an increasing degree of proficiency, these surgeons received a decreasing degree of supervision by an experienced surgeon, while being assigned to cases with increasing surgical difficulty. Surgeons with a career volume of more than 90 pancreatic resections were classified experienced.

Surgeons were considered to have had frequent practice if they had performed at least three resections within the last 6 weeks. Surgeons with a caseload of at least three resections in more than 6 weeks were categorized as sporadically trained. A constant frequency of practice of three resections within 6 weeks would correspond to a surgeon volume of at least 26 resections per year.

### Outcome measures, risk adjustment and statistical analysis

Patient characteristics were analyzed descriptively according to categories of surgical experience and the frequency of practice in pancreatic surgery. Due to the database structure and the limited ability to reconstruct the post-discharge course of our early patients, we only used outcome measures that were recorded during the hospital stay. In-hospital mortality, defined as death before discharge, was studied as a primary outcome measure. Secondary outcomes were rates of grade B/C delayed gastric emptying (DGE), postpancreatectomy hemorrhage (PPH) and postoperative pancreatic fistula (POPF) according to the International Study Group for Pancreatic Surgery (ISGPS) definitions [14–16]. In addition, the rate of patients with a length of stay (LOS) > 14 days was analyzed. Categorical variables were expressed as whole numbers and proportions. To assess the impact of the surgeon’s level of experience and the surgeon’s frequency of practice on these outcome measures, logistic regression models were used. Upon the pooled data of the entire observation period, regression models were fitted for all patients. The models were constructed using variables for risk adjustment including age groups, sex, stage of surgical difficulty and year of treatment. The exact definitions of these variables are given in Table 1. Results from the multivariable analysis were presented as odds ratios (ORs) with corresponding 95% confidence intervals (CI). The level of statistical significance was set to 0.1. Analyses were conducted using STATA Version 11 (STATA Corp).

**Table 1:** Patient Characteristics According to Level of Experience and Frequency of Practice

	Beginner		Intermediate		Experienced		All Patients (*n* = 1281)
	Sporadic (*n* = 125)	Frequent (*n* = 96)	Sporadic (*n* = 256)	Frequent (*n* = 163)	Sporadic (*n* = 241)	Frequent (*n* = 400)	
Age Groups, n (%)							
< 50	31 (24.8%)	25 (26.0%)	68 (26.6%)	47 (28.9%)	57 (23.6%)	67 (16.7%)	295 (23.0%)
50–70	67 (53.6%)	48 (50.0%)	122 (47.6%)	77 (47.2%)	140 (58.1%)	205 (51.3%)	659 (51.4%)
≥ 70	27 (21.6%)	23 (24.0%)	66 (25.8%)	39 (23.9%)	44 (18.3%)	128 (32.0%)	327 (25.6%)
Gender, n (%)							
Female	61 (48.8%)	39 (40.6%)	106 (41.4%)	69 (42.3%)	103 (42.7%)	156 (39.0%)	534 (41.7%)
Male	64 (51.2%)	57 (59.4%)	150 (58.6%)	94 (57.7%)	138 (57.3%)	244 (61.0%)	747 (58.3%)
Surgical Difficulty, n (%)							
Stage 1	21 (16.8%)	20 (20.8%)	18 (7.0%)	18 (11.0%)	31 (12.9%)	40 (10.0%)	148 (11.8%)
Stage 2	8 (6.4%)	5 (5.2%)	21 (8.2%)	11 (6.7%)	18 (7.5%)	27 (6.8%)	90 (7.0%)
Stage 3	78 (62.4%)	52 (54.2%)	189 (73.8%)	119 (73.0%)	151 (62.7%)	262 (65.5%)	851 (66.4%)
Stage 4	18 (14.4%)	19 (19.8%)	28 (10.9%)	15 (9.2%)	41 (17.0%)	71 (17.8%)	192 (15.0%)
Year of Surgery, n (%)							
1993–2000	53 (42.4%)	24 (25.0%)	63 (24.6%)	39 (23.9%)	66 (27.4%)	69 (17.2%)	314 (24.5%)
2001–2005	16 (12.8%)	25 (26.0%)	118 (46.1%)	36 (22.1%)	38 (15.8%)	71 (17.8%)	304 (23.7%)
2006–2009	26 (20.8%)	17 (18.0%)	50 (19.5%)	71 (43.6%)	60 (24.9%)	95 (23.8%)	319 (24.9%)
2010–2013	30 (24.0%)	30 (31.0%)	25 (9.8%)	17 (10.4%)	77 (31.9%)	165 (41.2%)	344 (26.9%)

Stage 1: Distal Pancreatectomy, Enucleation; Stage 2: Duodenum Preserving Pancreatectomy, Total Pancreatectomy; Stage 3: Proximal Pancreatectomy, Segmental Pancreatectomy; Stage 4: Additional Organ Resection

## Results

### Patient characteristics

In the study period of 1993 to 2013, 1335 patients underwent pancreatic resections. Excluded from this cohort were 54 patients (4%) who had incomplete or missing hospital data files due to a flooding of the hospital in 2002. Therefore, a total of 1281 patients were included in the final analysis. There were 25 operating surgeons. As the experience of the surgeons progressed over time, some surgeons were listed in more than one category at least once during the study period.

Overall mortality and overall cumulative morbidity were 3.7 and 22.2%, respectively. Of all resections, experienced surgeons (career volume greater than 90 resections) conducted 50% (*n* = 641), surgeons of intermediate experience 32.7% (*n* = 419) and pancreatic surgery novices 17.3% (*n* = 221), respectively (Table 1). We have previously shown that pancreatic surgery patients aged 70 and older have a higher in-hospital mortality rate than younger patients [17]. In this study, the proportion of patients aged 70 and older was higher in the experienced category compared with intermediate and novice categories (26.8% vs. 25.1% vs. 22.6%). 81.4% of all resections were of increased surgical difficulty (stage 3 and 4). Surgeons with experienced and intermediate expertise performed proportionally more resections with increased surgical difficulty (stage 3 and 4) compared to the novice surgeons (81.9% vs. 83.8% vs. 75.6%).

The frequencies of patient characteristics stratified by the level of surgical experience and frequency of practice are given in Table 1.

### Impact of surgical experience

The unadjusted rates of patients with a LOS of more than 14 days were 48.9, 48.4 and 47.1% for the novice, intermediate and experienced category, respectively. The unadjusted rates of cumulative morbidity (grade B/C POPF, PPH and DGE) and mortality were lower in the novice category (17.7 and 2.7%, respectively) as compared to the intermediate (23.9 and 5.5%) or experienced categories (22.6 and 2.8%). All unadjusted rates are given in Table 2.

**Table 2:** Crude Rates of Length of Stay (> 14 Days), Postoperative Morbidity and Mortality According to Level of Experience and Frequency of Practice

	Beginner		Intermediate		Experienced		All Patients (*n* = 1281)
	Sporadic (*n* = 125)	Frequent (*n* = 96)	Sporadic (*n* = 256)	Frequent (*n* = 163)	Sporadic (*n* = 241)	Frequent (*n* = 400)	
LOS > 14 Days, n (%)	60 *(48.0%)*	53 *(55.2%)*	137 *(53.5%)*	79 *(48.5%)*	134 *(55.6%)*	205 *(51.2%)*	668 *(52.1%)*
DGE, n (%)	14 *(11.2%)*	5 *(5.2%)*	30 *(11.7%)*	12 *(7.4%)*	25 *(10.4%)*	36 *(9.8%)*	122 (*9.5%)*
POPF, n (%)	10 *(8.0%)*	10 *(10.4%)*	39 *(15.2%)*	21 *(12.9%)*	26 *(10.8%)*	49 *(12.3%)*	155 *(12.1%)*
PPH, n (%)	9 *(7.2%)*	2 *(2.1%)*	26 *(10.2%)*	10 *(6.1%)*	24 *(10.0%)*	32 *(8.0%)*	103 *(8.0%)*
Cumulative Morbidity, n (%)	25 *(20%)*	14 *(14.6%)*	69 *(27.0%)*	31 (*19.0%)*	56 *(23.2%)*	89 *(22.3%)*	284 *(22.2%)*
In-hospital Mortality, n (%)	6 *(4.8%)*	0 *(0.0%)*	16 *(6.3%)*	7 (*4.3%)*	9 *(3.7%)*	9 *(2.3%)*	47 *(3.7%)*

Cumulative Morbidity Includes all Cases With Incidence of Grade B/C delayed gastric emptying (DGE), postoperative pancreatic fistula (POPF) and/or postpancreatectomy hemorrhage (PPH)

*LOS* Length of stay

Multivariate regression with adjustment for age, gender, surgical difficulty and year of surgery showed that the risk of LOS and DGE was not significantly associated to the level of surgical experience (Table 3). Compared to intermediate experience, the novice category was associated with a reduced risk of POPF (OR 0.46; 95% CI 0.26–0.82), PPH (OR 0.51; 95% CI 0.25–1.05) and in-hospital mortality (OR 0.45; 95% CI 0.17–1.16). The experienced category was associated with a reduced risk of POPF (OR 0.54; 95% CI 0.36–0.82) and in-hospital mortality (OR 0.42; 95% CI 0.21–0.83) compared to the level of intermediate experience.

**Table 3:** Risk-adjusted Odds Ratios of Length of Stay, Morbidity and In-hospital Mortality According to the Surgeon’s Level of Experience

	Level of Experience	Risk-adjusted OR	95% CI
LOS > 14 Days	Beginner	0.98	0.69–1.39
	Intermediate	1.00	Reference
	Experienced	0.97	0.74–1.27
DGE*	Beginner	0.82	0.45–1.50
	Intermediate	1.00	Reference
	Experienced	0.72	0.46–1.13
POPF*	Beginner	0.46	0.26–0.82
	Intermediate	1.00	Reference
	Experienced	0.54	0.36–0.82
PPH*	Beginner	0.51	0.25–1.05
	Intermediate	1.00	Reference
	Experienced	0.85	0.53–1.37
In-hospital Mortality	Beginner	0.45	0.17–1.16
	Intermediate	1.00	Reference
	Experienced	0.42	0.21–0.83

Odds ratios were calculated by multivariate regression with adjustment for age, gender, surgical difficulty and year of surgery. *Includes Grade B + C

*DGE* delayed gastric emptying, *POPF* postoperative pancreatic fistula, *PPH* postpancreatectomy hemorrhage, *LOS* Length of stay (LOS)

### Impact of Surgeon’s frequency of practice

Surgeons with frequent operating exposure had lower observed rates of cumulative morbidity and in-hospital mortality (Table 2). Independent from the level of experience, frequency of practice was associated with a significantly lower risk of DGE (OR 0.56, 95% CI 0.38–0.83), PPH (OR 0.64, 95% CI 0.42–0.98) and in-hospital mortality (OR 0.45, 95% CI 0.24–0.87) (Table 4).

**Table 4:** Risk-adjusted Odds Ratios of Length of Stay, Morbidity and In-hospital Mortality according to the Surgeon’s Frequency of Practice

	Frequency of Practice	Risk-adjusted OR	95% CI
LOS > 14 Days	Sporadic	1.00	Reference
	Frequent	0.89	0.70–1.12
DGE*	Sporadic	1.00	Reference
	Frequent	0.56	0.38–0.83
POPF*	Sporadic	1.00	Reference
	Frequent	0.88	0.62–1.24
PPH*	Sporadic	1.00	Reference
	Frequent	0.64	0.42–0.98
In-hospital Mortality	Sporadic	1.00	Reference
	Frequent	0.45	0.24–0.87

Odds ratios were calculated by multivariate regression with adjustment for age, gender, surgical difficulty and year of surgery. *Includes Grade B + C

*DGE* delayed gastric emptying, *POPF* postoperative pancreatic fistula, *PPH* postpancreatectomy hemorrhage, *LOS* Length of stay (LOS)

Upon the combined inclusion of surgical experience and frequency of practice into our regression models, we found a significantly reduced risk of DGE for frequently trained novice, intermediate and experienced surgeons compared to sporadic trained intermediates (Table 5). We also detected significantly reduced ORs of POPF in sporadic and frequently trained novices and experienced surgeons compared to sporadic trained intermediates. In addition, the category of frequently trained experienced surgeons was associated with significantly reduced rates of in-hospital mortality, whereas frequently trained novices had significantly reduced rates of PPH. There was no mortality in the category of frequently trained beginners, which did not allow for calculation of OR.

**Table 5:** Risk-adjusted Odds Ratios of Length of Stay, Morbidity and In-hospital Mortality according to the Surgeon’s Level of Experience and Frequency of Practice

	Level of Experience	Frequency of Practice	Risk-adjusted OR	95% CI
LOS > 14 Days	Beginner	Sporadic	0.78	0.49–1.24
		Frequent	1.19	0.72–1.96
	Intermediate	Sporadic	1.00	Reference
		Frequent	0.89	0.58–1.34
	Experienced	Sporadic	1.12	0.76–1.64
		Frequent	0.82	0.58–1.16
DGE*	Beginner	Sporadic	0.97	0.47–2.01
		Frequent	0.33	0.12–0.92
	Intermediate	Sporadic	1.00	Reference
		Frequent	0.54	0.26–1.12
	Experienced	Sporadic	0.71	0.39–1.31
		Frequent	0.49	0.28–0.87
POPF*	Beginner	Sporadic	0.42	0.19–0.89
		Frequent	0.47	0.22–1.02
	Intermediate	Sporadic	1.00	Reference
		Frequent	0.87	0.48–1.58
	Experienced	Sporadic	0.52	0.29–0.92
		Frequent	0.51	0.31–0.85
PPH*	Beginner	Sporadic	0.68	0.30–1.55
		Frequent	0.16	0.04–0.74
	Intermediate	Sporadic	1.00	Reference
		Frequent	0.65	0.30–1.42
	Experienced	Sporadic	0.89	0.48–1.67
		Frequent	0.64	0.35–1.15
Mortality	Beginner	Sporadic	0.79	0.29–2.21
		Frequent	no deaths	
	Intermediate	Sporadic	1.00	Reference
		Frequent	0.80	0.31–2.07
	Experienced	Sporadic	0.56	0.23–1.38
		Frequent	0.29	0.12–0.71

Odds ratios were calculated by multivariate regression with adjustment for age, gender, surgical difficulty and year of surgery. *Includes Grade B + C

DGE - delayed gastric emptying, POPF - postoperative pancreatic fistula, PPH - postpancreatectomy hemorrhage, LOS - Length of stay (LOS)

Notably, inclusion of frequency of practice seemed to enforce the effect of the level of experience on DGE, POPF and in-hospital mortality, when the frequently trained novice and experienced surgeons were compared to rarely trained intermediates (e.g. experienced surgeons: ORexperience: 0.42 vs. ORexperience and frequency of practice: 0.29).

## Discussion

Today, the way we consider surgical training has changed. Due to the rapid evolution of new techniques, the apprenticeship model is in desperate need of augmentation. The overwhelming evidence supporting the transfer of skills from the simulation laboratory to the operating room has led to the acceptance of simulation training in surgery. This, in turn, enabled research into educational concepts for technical skills previously described in domains outside medicine, such as deliberate practice and mental practice [18]. However, in the foreseeable future supervised hands-on training will remain a critical part of training in complex surgery. Therefore, in addition to new educational concepts and innovations in simulation, a better understanding of associations between training status and patient outcomes is needed to improve the classical training model. Our study demonstrates that pancreatic surgery training with stringent case selection and intensive supervision enables trainee surgeons to achieve perioperative outcomes comparable to those of experienced surgeons. These findings are supported by studies that have already implied that supervised trainees do not jeopardize patient outcomes in pancreatic surgery [13, 19]. Nevertheless, it appears reasonable that an increasing trainee autonomy may be associated with higher complications rates. Notably, our study shows that reduction of supervision and less stringent case selection in the intermediate level impair outcomes. Moreover, there are other studies that revealed learning curves in resident and fellowship training [12, 20], which is also in line with our data showing a significant improvement of outcomes between the intermediate and experienced categories. In view of these data we believe that there is a transitional period between surgical training and the gaining of profound expertise that is associated with impaired perioperative results. In this regards, adequate case selection and prolonged supervision may be recommended even for surgeons that are competent to perform pancreatic resections on their own but are still within their learning curve (= intermediate level).

Numerous studies have identified annual surgeon caseload as significant a independent variable of death following pancreatic surgery [5, 21, 22]. Instead of using surgeon volume per year, we applied a novel concept using a short time interval of 6 weeks and a threshold of at least three resections to differentiate between sporadic and frequent practice in the short term. This definition ensured that surgeons meeting these criteria had constantly trained or repeated pancreatic resections in advance of the procedure of interest. Our analysis shows that frequent practice improves rates of in-hospital mortality and pancreatic surgery – specifically morbidity. It is worth noting that this effect is present in every level of surgical experience. To our knowledge, this is the first study examining the frequency of practice in the short term, thereby showing that frequent repetitions of a procedure have a significant impact on perioperative morbidity independent from the level of experience. We conclude from these results that training programs for pancreatic surgery should be reserved to institutions having the necessary patient caseload.

This study has several limitations that need to be appropriately taken into consideration. Firstly, although the data were extracted from a prospective database, this was a retrospective study with all of the associated bias risks. In this regard, the distribution of cases between novice and expert surgeons have probably been subject to a selection bias. Secondly, our database encompasses only data from a single institution, possibly limiting the ability for the broad generalization of our findings to all institutions with a training program for pancreatic surgery. Thirdly, with increasing proficiency, intermediately experienced surgeons were likely to be assigned to cases with increasing surgical difficulty. We, therefore, ranked surgical procedures according to the stage of difficulty and added this variable into our regression models. Lastly, we were unable to determine the amount of preexisting hands-on experience in general surgery of pancreatic surgery trainees. Due to the increasing trend of sub-specialization, today’s trainees may have less operative exposure when performing their first pancreatic resection compared to their predecessors. In addition, changes of practice may have occurred during the analysed time period of 20 years. We, therefore, included the year of surgery into our multivariate regression models to reduce the confounding effect of time-related changes.

## Conclusions

Our study demonstrates that surgical training with intensive supervision ensures patient safety, whereas the reduction of supervision based upon the progression of surgical competency may have unfavorable effects on surgical outcomes. These results emphasize the importance of supervision of surgeons that are competent to perform pancreatic resections on their own but are still within their learning curve. The fact that frequent practice improves perioperative outcomes in every level of experience underlines the need of a sufficient patient caseload to ensure frequent hands-on practice.

## Data Availability

The datasets used and analysed during the current study are available from the corresponding author on reasonable request.

## Appendix

**Table 6:** Definition of the Surgeon’s Training Status

Training Status	Level of Experience (Total No. of Pancreatic Resections and Supervision)		Frequency of Practice (Last 3 Pancreatic Resections)
Beginner - Sporadic	< 20	Intensive	> 6 Weeks
Beginner - Frequent	< 20	Intensive	≤ 6 Weeks
Intermediate - Sporadic	21–90	Decreasing	> 6 Weeks
Intermediate - Frequent	21–90	Decreasing	≤ 6 Weeks
Experienced - Sporadic	≥ 90	None	>6 Weeks
Experienced - Frequent	≥ 90	None	≤ 6 Weeks

## Abbreviations

CI: Confidence interval
DGE: Delayed gastric emptying
LOS: Length of stay
OR: Odds ratio
POPF: Postpoperative pancreatic fistula
PPH: Postoperative pancreatic hemorrhage
